# Encouraging Patient Portal Use in the Patient-Centered Medical Home: Three Stakeholder Perspectives

**DOI:** 10.2196/jmir.6488

**Published:** 2016-11-22

**Authors:** Gemmae M Fix, Timothy P Hogan, Daniel J Amante, D Keith McInnes, Kim M Nazi, Steven R Simon

**Affiliations:** ^1^ Center for Healthcare Organization and Implementation Research Bedford, MA United States; ^2^ Department of Health Law, Policy, and Management Boston University School of Public Health Boston, MA United States; ^3^ Division of Health Informatics and Implementation Science, Department of Quantitative Health Sciences University of Massachusetts Medical School Worcester, MA United States; ^4^ Veterans Health Administration Veterans and Consumers Health Informatics Office, Office of Connected Care US Department of Veterans Affairs Washington, DC United States; ^5^ Section of General Internal Medicine VA Boston Healthcare System Boston, MA United States

**Keywords:** personal health record, portal, enrollment, primary care, patient-centered medical home, qualitative

## Abstract

**Background:**

Health care organizations are increasingly offering patients access to their electronic medical record and the ability to communicate with their providers through Web-based patient portals, thus playing a prominent role within the patient-centered medical home (PCMH). However, despite enthusiasm, adoption remains low.

**Objective:**

We examined factors in the PCMH context that may affect efforts to improve enrollment in a patient portal.

**Methods:**

Using a sociotechnical approach, we conducted qualitative, semistructured interviews with patients and providers from 3 primary care clinics and with national leaders from across a large integrated health care system.

**Results:**

We gathered perspectives and analyzed data from 4 patient focus groups and one-on-one interviews with 1 provider from each of 3 primary care clinics and 10 program leaders. We found that leaders were focused on marketing in primary care, whereas patients and providers were often already aware of the portal. In contrast, both patients and providers cited administrative and logistical barriers impeding enrollment. Further, although leadership saw the PCMH as the logical place to focus enrollment efforts, providers and patients were more circumspect and expressed concern about how the patient portal would affect their practice and experience of care. Further, some providers expressed ambivalence about patients using the portal. Despite absence of consensus on how and where to encourage portal adoption, there was wide agreement that promoting enrollment was a worthwhile goal.

**Conclusions:**

Patients, clinicians, and national leaders agreed that efforts were needed to increase enrollment in the patient portal. Opinions diverged regarding the suitability of the PCMH and, specifically, the primary care clinic for promoting patient portal enrollment. Policymakers should consider diverse stakeholder perspectives in advance of interventions to increase technology adoption.

## Introduction

### Background

Health care organizations are increasingly engaging patients in the management and coordination of their own care [1]. This patient-centered model of health care positions the patient as an integral member of the care team and allows for patients not only to receive information about their health, but also to contribute information that informs their care [2]. Information and communications technologies (ICTs) that facilitate the sharing and exchange of information between patients and their clinical teams’ members are a key aspect of patient-centered care. One such technology that equips patients with tools to interact with their clinical teams is the Web-based patient portal. In recent years, patient portals have evolved from providing patients with a way to view information in their medical record to also encompass secure Web-based apps that offer various electronic tools to support health care system transactions, information tracking, and communication [3]. Health care systems have increasingly promoted the use of patient portals, motivated in part by a desire to satisfy “meaningful use” requirements of the Medicare and Medicaid Electronic Health Record (EHR) Incentive program [4]. Most large health care organizations [5-7], including the Veterans Health Administration (VHA) [8,9], offer patient portals. Although the functionality of patient portals varies, all strive to increase patient engagement.

The use of ICTs such as patient portals is often considered a critical component of a patient-centered medical home (PCMH). The PCMH has been described as a way of organizing primary care that emphasizes coordination and communication, and better aligns primary care with patients’ goals [10]. As part of a broader transformational initiative to realize the principles of patient-centered care, the VHA broadly implemented a PCMH model beginning in 2010 [11,12]. In this model, every patient is assigned to a PCMH, which typically consists of a primary care provider, nurse, medical support assistant, and access to professional staff, such as clinical pharmacists, mental health specialists, social workers, or nutritionists, all who work collaboratively to care for a panel of patients [13]. Promoting patient engagement through the use of ICTs has been a key element in implementing the VHA’s PCMH model [14,15]. VHA’s patient portal, My Health*e*Vet, enables patients to view, print, and download information (eg, clinicians’ notes, laboratory results) from their EHR, communicate electronically with their health care team using secure messaging, refill prescriptions, view wellness reminders, and access educational information. See Multimedia Appendix 1 for a summary of PCMH principles and exemplary features of the My Health*e*Vet patient portal.

Despite the potential benefits associated with patient portal use and the role that they are envisioned to play in PCMH, the actual enrollment of patients has remained low [16,17]. A number of possible reasons contribute to low enrollment, including limited awareness [18], lack of familiarity with computers and the Internet [19], low levels of health literacy [20], and lack of provider endorsement [14]. At the time of this study, less than 1 in 5 Veterans using VHA health care had enrolled in My Health*e*Vet. Currently, more than half of VHA patients now access My Health*e*Vet; however, challenges remain.

### Study Goals

Given the central role of PCMH in many health care systems, including the VHA, PCMH settings may be an ideal place to reach patients and increase enrollment in patient portals. Understanding the potential of the PCMH setting to enroll patients in a patient portal requires an in-depth understanding of influential contextual factors [21]. Similarly, a sociotechnical perspective emphasizes the need to examine the interrelationship between technology and its social environment [22,23]. As such, our objective was to gather the perspectives of 3 different stakeholder groups to understand the range of sociotechnical factors affecting efforts to improve enrollment in the My Health*e*Vet patient portal.

## Methods

### Study Design, Setting, and Participants

Our qualitative study design used focus groups and semistructured interviews to ascertain 3 critical perspectives: patients, primary care team providers, and program leaders. The patient and provider components of the study took place in 3 primary care clinics at 2 VHA Medical Centers in the northeast United States in 2011 and 2012. Program leaders included VHA employees who served on national working groups that guided the development of and set policy for VHA patient portal use. Informed consent was obtained from all participants. VHA employees were not compensated; patients received US $20. Study procedures were approved by the appropriate VHA Institutional Review Boards.

At the time of the study, enrollment in My Health*e*Vet required multiple steps. First, patients needed to establish an account through an online registration process. Second, patients were required to visit a VHA facility to verify their identity; this process is known as in-person authentication. Third, patients wishing to use the secure messaging feature of My Health*e*Vet had to “opt in” in an additional step. Thus, we identified 4 classes of patients: (1) not registered for My Health*e*Vet, (2) registered but not yet have in-person authentication, (3) had in-person authentication but did not opt in for secure messaging, and (4) opted in to secure messaging. The first group was considered “not enrolled” for the purposes of this research; the 3 other classes were considered “enrolled.”

### Data Collection

We employed a convenience sampling strategy to recruit patients, providers, and leadership.

#### Patient Focus Groups

Patients were recruited using flyers posted and pamphlets handed out in primary care. VHA databases were used to identify participants to recruit by mail. We held focus groups for “enrolled” and “not enrolled” patients using the preceding criteria.

We held 4 focus groups: 2 for enrolled patients, 2 for unenrolled patients. The focus group guides (see Multimedia Appendix 2) were similar, but tailored to enrollment status. All focus group participants were asked about familiarity with the patient portal, practices for managing health information, and computer familiarity and use. Participants were also asked about receptivity to learning about the My Health*e*Vet patient portal in the primary care setting and strategies to increase enrollment. For patients who had already enrolled in the portal, we also asked about their experience in enrolling. Data were analyzed according to enrollment status to discern if there were differences between these groups. Focus groups were held in private rooms near the primary care setting and were audio recorded. Each lasted approximately one hour.

#### Provider and Program Leader Interviews

Providers were identified by primary care clinic affiliation and recruited in person and via email. Program leaders were identified by their national, system-wide role in the My Health*e*Vet patient portal program and subsequently recruited by email and telephone. Participants included individuals who served on policy-making committees. Others were involved in the design and evaluation of the patient portal; some were active in clinical roles in their local VHA Medical Centers. For the provider interviews, we developed a semistructured interview guide to assess clinicians’ familiarity with the My Health*e*Vet patient portal, experiences discussing the portal with patients, and their perceptions of patient interest and portal use among their patients. For the program leaders, we developed a semistructured interview guide to elicit the history of the My Health*e*Vet patient portal, understand existing efforts to improve enrollment practices in primary care as well as other settings, gain feedback on potential enrollment interventions, and understand the evolution of the portal. Interviews were conducted over the telephone or in person and audio recorded with permission. Each interview lasted approximately 30 minutes.

Both the focus group and interview guides were developed through iterative rounds of review by the team. They were designed to be used flexibly and tailored to the group or unique position of each interviewee.

### Analysis

Focus group and interview data were transcribed verbatim. In an effort to maximize rigor and trustworthiness, we engaged multiple team members in our analysis who met regularly and coded the transcripts using emergent analytic techniques involving a grounded theory approach [24]. Initially, team members GF, DA, and TH each independently reviewed a transcript reflecting each of the 3 stakeholder groups in the study and then met to compare their respective findings. The outcome of this meeting was a codebook that was applied to all transcripts in iterative rounds of analysis. As coding proceeded, clinicians and program evaluators with deep knowledge of VHA’s primary care context and the My Health*e*Vet patient portal were consulted and asked to provide feedback on the team’s analytic interpretations. Coding was performed in Microsoft Word, using separate documents to capture text exemplifying codebook themes. This process was initially done separately for the different patient focus groups, and provider and leadership interviews. Subsequently, we synthesized themes across the groups.

## Results

We conducted 4 patient focus groups and interviewed 1 primary care provider from each of the 3 clinics, along with 10 program leaders. 5 key themes that cut across the data were identified:

1. Disconnect over the role of marketing in primary care to increase enrollment;

2. Differing perspectives on where barriers to enrollment exist;

3. Divergence of opinions on the appropriateness of primary care for promoting personal health record (PHR) portal enrollment;

4. Provider ambivalence regarding the value of the My Health*e*Vet PHR portal; and

5. Lack of consensus over appropriate patients to target for My Health*e*Vet PHR portal enrollment.

### Disconnect Over the Role of Marketing in Primary Care to Increase Enrollment

Leadership was focused on the potential of marketing to increase awareness and enrollment, whereas providers and patients felt saturated with information about the My Health*e*Vet PHR portal. The program leader interviews centered on ways to promote and market My Health*e*Vet:

If we start the marketing perspective I think there is a lot more that can get involved before getting to the registration piece. I think pharmacy techs, lab techs, volunteers at the front desk, they could all be involved in the marketing, handing out a flyer.

Leader

In contrast, the provider interviews did not emphasize patients’ awareness, focusing instead on identifying which patients are most likely to be interested in My Health*e*Vet. In response to a question about raising My Health*e*Vet with his patients, one provider shifted the conversation to discuss which patients are appropriate for My Health*e*Vet:

We’ve been aggressively trying to engage our patients to sign up for My HealthVet and [secure messaging]; however, I do believe that difference in the demographics and patient population has its bearing on how well it happens. It happens still that this particular practice tends to be more geriatric with less computer savviness.

Provider

This same provider was asked if patients ever bring up My Health*e*Vet. He responded, *“* Usually by the time they get to my office, they are fully aware of the existence of this as an option, and they don’t need me as an advisor for computer training options here.”

The patients we spoke with, including those who were not yet enrolled in My Health*e*Vet, were aware of My Health*e*Vet. Patients described learning about My Health*e*Vet through a variety of sources including providers, other patients, and promotional materials such as online advertisements, posters, mailed materials, and brochures handed out during appointments. Not all patients viewed the brochures favorably:

I’m sure I have probably about 40 copies of this [My HealthVet brochure]...But because I have this [brochure], I have that [brochure], I have this, I have that...by the time I get home, it’s like, “Take all this [VHA information] and chuck it. Put it in the recycle bin.”

Unenrolled patient

Similarly, a program leader lamented that marketing materials, such as water bottles and lanyards with the My Health*e*Vet logo, were not utilized at the anticipated rate. Further, he noted the importance of being persistent but also making sure not to tell the same patient repeatedly about My Health*e*Vet: “We have to be a little bit careful when a [patient] says, ‘No, I’m not interested, don’t ask me again,’ we have to make sure that we don’t ask them again.”

### Differing Perspectives on Where Barriers to Enrollment Exist

Discussions in patient focus groups repeatedly returned to issues about challenges to completing enrollment, which were less prominent in leadership interviews. The providers we spoke with recounted administrative and logistical problems with the My Health*e*Vet enrollment process that they encountered in their primary care practices. Likewise, much of the patient focus groups, both the enrolled and unenrolled, were spent discussing problems the participants encountered in trying to enroll. One unenrolled patient stated, “The steps we have to go through to register [are] just ridiculous.” Notably, more than half (7/12) of the unenrolled focus group participants reported having tried to enroll in My Health*e*Vet. Several of these participants thought they had completed all the steps necessary to access the full range of My Health*e*Vet features. One was certain she was fully registered, despite the research team identifying her as unenrolled from verified databases. Another said her provider told her that she was registered, but she still reported that she could not access My Health*e*Vet.

**Figure 1:**
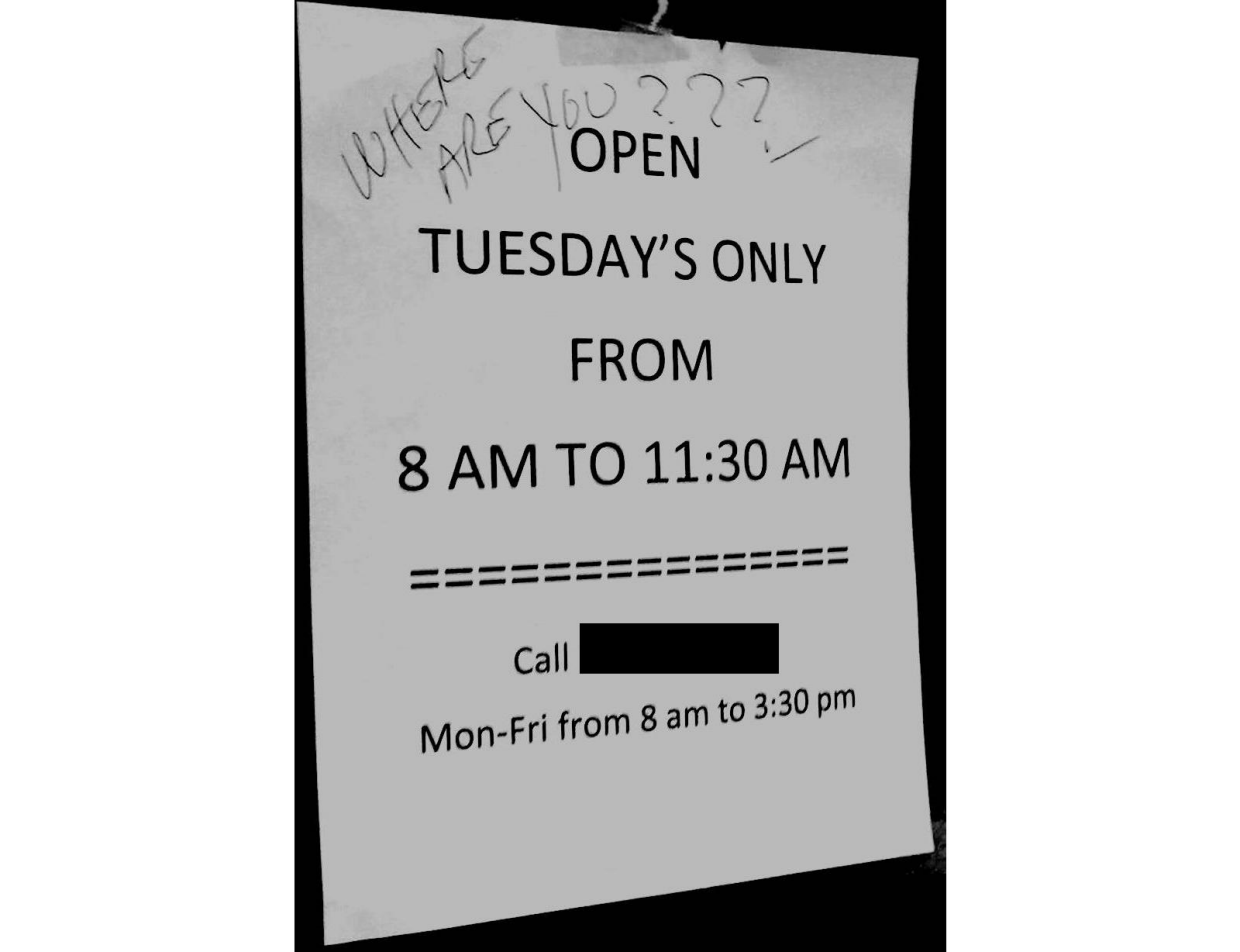
Sign on door to My Health*e*Vet enrollment office at one of the study sites.

Many of the patients in the “enrolled” focus groups had similar experiences to those in the “unenrolled” groups, especially when describing challenges in completing the enrollment process. In-person authentication was particularly problematic. At the medical centers where the study was conducted, in-person authentication was available in 1 location. Patients reported that the office was difficult to find and had limited hours (see Figure 1): “There is an office downstairs, they tell me to go into and sign up, every time I go to that office, it’s closed” (Unenrolled patient).

Providers were aware of patients’ logistical difficulties trying to enroll in My Health*e*Vet. One provider characterized the current enrollment process as “completely out of touch [with] reality” because patients—who may have taken time off of work to come to their clinical appointment—were expected to go to another location in the hospital, sometimes on a different day, to enroll in My Health*e*Vet. This physician described problems with the location and hours of the in-person authentication office:

The part that bums me is how many [patients] have gone to that office, saw it was closed and never let me know, and just months went by until the next visit, and they said, “yeah, you know, I’ve tried to go in there and it was closed.”

Provider

Of note, this clinician subsequently told her patients how to bypass the official enrollment office. She referred patients to a different office, which had more regular hours and staff willing to help patients complete the enrollment.

In contrast, in interviews with national program leaders, issues related to logistical barriers were not brought up, aside from one leader referring to a potential enrollment “glitch” that might prevent a patient from using My Health*e*Vet, during a larger discussion about the importance of getting providers to use secure messaging.

### Divergence of Opinions on the Appropriateness of Primary Care for Promoting Personal Health Record Portal Enrollment

Leadership assumed primary care was the best and most logical location within the organization for promoting PHR portal enrollment; however, providers and patients preferred to focus on pressing clinical issues. Program leaders saw a role for primary care providers in the patient portal enrollment process. Although they did not feel that providers needed to be directly involved, they nonetheless felt that providers needed to play a strong supporting role by both encouraging their patients to enroll and supporting coworkers’ efforts to enroll patients, in keeping with the PCMH model:

Physicians, they have to champion it. That is going to be a critical piece. If the physician doesn’t champion it, then nobody else is going to get behind it.

Leader

The program leaders were aware of providers’ concerns about the potential workload involved in promoting and enrolling one’s patients in My Health*e*Vet. One noted the importance of ensuring that providers did not perceive My Health*e*Vet enrollment “as yet another thing to review with Veterans.”

The program leaders uniformly acknowledged that primary care providers had limited time to personally enroll patients in My Health*e*Vet: “The clinicians, the health care team members play a role, and they play an important role, but the scope of that role needs to be limited...It’s got to be a group effort.”

Yet, the program leaders still felt the provider role was a critical part of the process: “It’s fine to have the nurse do it, but I would also argue that the physician should also be the one saying [to their coworkers], ‘You need to do this.’”

In contrast, providers viewed My Health*e*Vet promotion less as a shared responsibility and more as needing to be the responsibility of other team members.

At the time of our fieldwork, the 3 primary care clinics serving as study sites had instituted a My Health*e*Vet patient portal clinical reminder in the EHR. The reminder would appear for primary care patients, as part of a series of wellness reminders that primary care clinicians and staff were responsible for resolving in the EHR. Even though providers and support staff all saw the My Health*e*Vet clinical reminder, the providers we spoke with viewed the reminder as “something for the medical assistants, the [licensed practical nurses], whoever is doing the intake of the patient. I don’t see that as my reminder, so I don’t act on it.” Another provider similarly stated, “I also don’t think it should be a physician-driven reminder; it should be filled by someone else on the team.” Moreover, one provider thought primary care should have a limited role in My Health*e*Vet enrollment, and that it should instead be a broader, system-level responsibility: “I think if [getting patients enrolled is] going to work it needs to—it’s an institutional issue, it’s not, I don’t see it as necessarily as a primary care issue.”

The providers we spoke with were largely uninterested in being involved in discussions with patients about My Health*e*Vet. One stated, “I do not have time in my practice to advocate for My Health*e*Vet use routinely.”

Moreover, the patients we spoke with felt that the primary care setting was not the appropriate place for patients to learn about My Health*e*Vet. They felt their primary care team members—including not only their provider, but also the receptionist, medical support assistant, and nurse—were too busy to talk to patients about My Health*e*Vet. The focus group participants did not want to receive information about My Health*e*Vet during their clinical appointments. They already felt they received considerable informational materials while in the primary care clinic. For My Health*e*Vet materials in particular, they felt that it was incongruous to include this information along with brochures about cholesterol and influenza vaccines. Further, when providers brought My Health*e*Vet up in the context of other clinical discussions, patients were confused:

They gave me the My HealthVet paperwork...and just a brief overview, but then at the same time they’re giving me information about cholesterol...I feel overwhelmed...I really kind of didn’t get My HealthVet.

Unenrolled patient

Some patients felt uncomfortable with their primary care provider promoting My Health*e*Vet during an appointment. One participant thought her provider had been too assertive in saying, “This is the *only* way you can communicate with me!”

Providers, too, were generally unenthusiastic about the My Health*e*Vet clinical reminders:

We have so many reminders that just get read in a robotic way, that it may just be noise to the patient, and if the person delivering the information isn’t excited or truly on board with the process, I don’t think it’s, it’s going to be useful.

Provider

Another commented:

There are other times when half the reminders don’t get done, and the ones that are done, the patient had no clue that they were done, so it raises some concern in my mind that the communication between the [medical assistant] and the patient is not very effective.

Provider

Beyond primary care and its PCMH model, the program leaders we spoke with felt that others in the medical center needed to be responsible for promoting My Health*e*Vet. They emphasized that leadership throughout each medical center should participate in My Health*e*Vet. In addition to local leadership support, the national program leaders felt other clinical services should share the responsibility with primary care for My Health*e*Vet enrollment: “We should also be having the lab[oratory employees] telling people that they can get the results of their blood tests [through My Health*e*Vet].”

### Provider Ambivalence Regarding the Value of the My Health *e* Personal Health Record Portal

Although leadership saw clear value in the use of the My Health*e*Vet PHR portal, providers were less convinced of its utility in practice. National leaders felt provider buy-in was key to promoting My Health*e*Vet. One program leader stressed that local leaders need to “both model that I’m using My Health*e*Vet and demonstrate some basic knowledge of how to use it, really advocate for it” and “not be cynical.” Yet all 3 participant groups—patients, providers, and program leaders—acknowledged that there was some provider ambivalence. Patients reported that although some providers aggressively encouraged enrollment, others seemed indifferent or even negative about My Health*e*Vet. Providers expressed mixed feelings. Some appreciated that My Health*e*Vet made medication refills easy for patients and subsequently reduced workload. However, other providers expressed concerns about My Health*e*Vet, from how it might affect patient-provider relationships to what information in the medical record their patients would be able to see. Others had concerns about enrolling patients in a system that they perceived as not fully functional. One of the providers was uneasy about the upcoming option for patients to view the clinician’s progress notes, which VHA added in 2013:

I’m also concerned about the fact that patients will see full progress notes. To the extent that patients start reading their own medical record directly—I would say that there is nothing in my note that should be offensive to a patient. But if a patient has problems with compliance, if they have problems with substance abuse, if we feel they’re manipulating and we need to communicate that to keep track of that ourselves, and communicate that to each other.

Provider

This provider went on to elaborate concerns about patients viewing documentation in the medical record that he viewed as primarily intended to communicate to other clinicians about sensitive matters, such as substance abuse or poor adherence.

In contrast, another provider, who had previously worked in a different health care system which had for several years been using a patient portal, said she promotes My Health*e*Vet use because of the ability to exchange secure messages with her patients. She saw the secure messaging feature as especially useful because the alternative was having patients use the telephone call center. She described the call center as unreliable; she did not consistently receive patients’ messages. In contrast, when her patients used secure messaging, no communications were lost.

### Lack of Consensus Over Appropriate Patients to Target for My Health *e* Personal Health Record Portal Enrollment

Primary care providers relied on their beliefs about who they thought might be appropriate for My Health*e*Vet use to guide conversations about enrollment, whereas leaders felt it should be targeted to all patients with computers. Providers did not think My Health*e*Vet was appropriate for all patients. One felt she had a good sense of her patients’ receptivity to My Health*e*Vet. She demonstrated this to the interviewer by reviewing her patient list for the day and commenting on each patient’s likelihood of using My Health*e*Vet. She cautioned that being older should not be seen as an exclusion criterion and went on to describe her octogenarian father’s extensive computer use. This view was not shared by other providers. Another said that in addition to age, computer literacy was an issue:

The main barrier, at least in this practice, is the fact that it is geriatric population. Even if it wasn’t a geriatric population, with my younger patients, it’s a question of computer literacy.

Provider

Some providers promoted My Health*e*Vet to patients who were younger or showed interest in computers. Other providers were more passive, only bringing up My Health*e*Vet in response to patients who showed an interest in technology or inquired about My Health*e*Vet:

There have been a couple of times with younger patients who I know use computers that I may have mentioned it, and asked them if they got the My HealthVet information, and encourage them to sign on, but I don’t do that with the majority of my patients.

Provider

This provider went on to say, “I wait for clues that the patient has some interest. My approach is to reinforce them, rather than be proactive, and saying, ‘This is on my checklist to make sure [you enroll].’”

Even a provider who described himself as highly supportive of My Health*e*Vet responded: “I don’t have really in-depth conversations with people who don’t indicate with me that they would want to use it.”

A focus group participant in his 80s noted that his providers had not mentioned My Health*e*Vet. Another described seeing My Health*e*Vet promotional materials, but initially thought My Health*e*Vet was for younger patients. He described how his clinician mentioned that My Health*e*Vet would allow him to have direct communication without using the telephone. This patient noted that as a result of this interaction, his perception changed—he realized that My Health*e*Vet was not limited to younger patients.

Instead of targeting My Health*e*Vet based on demographics, national leaders spoke of tailoring My Health*e*Vet promotion to patients with Internet access, such as having the clinical reminder begin by asking about access to the Internet. If the patient reported no access, they would no longer be targeted for enrollment.

## Discussion

In this qualitative study, we sought to identify and understand factors in VHA’s primary care context that might affect enrollment in the health care system’s PHR portal. Through our discussions with 3 stakeholder groups—patients, providers, and leadership—we found differing views of both the value of the My Health*e*Vet PHR portal as well as whether primary care in its role as the medical home was an appropriate location to support portal enrollment. 5 salient themes representing these divergent views emerged from our analysis. We discuss each theme subsequently as well as the perspectives of stakeholders and how our findings align with, and add to, the existing literature.

### Disconnect Over the Role of Marketing in Primary Care to Increase Enrollment

The program leaders we spoke with differed from patients and providers on their perspective about the role of marketing to increase awareness. Although program leaders perceived that lack of awareness was a significant issue, which additional marketing could address, findings from both patients and providers suggested otherwise. The providers felt their patients were aware of My Health*e*Vet and that any marketing efforts needed to be tailored specifically to patients who were most likely to enroll. The patients we spoke with were generally aware of My Health*e*Vet, with several noting the abundance of marketing materials being distributed in primary care. However, patients were less familiar with *how* to enroll in My Health*e*Vet. Although the program leaders were focused on marketing strategies, our patient data suggest that knowledge of My Health*e*Vet is not the prominent barrier to enrollment. Instead, patients encountered difficulty with enrollment procedures. An enrollment strategy where patients are automatically enrolled and would have to opt out if they were not interested could vastly reduce patient enrollment burden [25]. Additionally, VHA has updated its marketing and outreach strategy utilizing social media, online YouTube videos, and partnering with community organizations [26].

### Differing Perspectives on Where Barriers to Enrollment Exist

The patient and provider interviews focused their discussions on their poor experiences with the My Health*e*Vet enrollment process. Patients in both the enrolled and unenrolled focus groups recounted similar barriers to enrolling, the notable difference being that the enrolled participants were ultimately successful. Moreover, it appeared that a number of patients who had begun the registration process had failed to complete all the necessary steps to gain access to valuable features of the portal, such as secure messaging and viewing laboratory results. Of those who were unenrolled, many were not aware they had not completed all the steps of the enrollment process. In contrast, this discussion of barriers was not a prominent theme in the leadership interviews. Instead, their focus was on marketing and increasing awareness at this early stage of the My Health*e*Vet initiative.

Since the completion of the study, PHR portals and strategies to engage patients to adopt and use them have continued to evolve. At VHA, several changes have occurred to improve awareness of and enrollment in My Health*e*Vet. Some VHA Medical Centers have established organizational structures outside of the primary care setting to support patients who are interested in learning more about My Health*e*Vet (eg, establishing a special group visit clinic). These settings also provide patients with assistance in completing the enrollment process and often offer educational opportunities to learn how to use the various portal tools effectively [27,28]. Other sites have successfully tested providing clinic clerks with prompts and resources to engage with patients about interest in completing My Health*e*Vet enrollment as part of the initial enrollment process for VHA services. This innovation is now being implemented within the EHR nationwide. Many VHA Medical Centers also offer My Health*e*Vet enrollment via point-of-service kiosks. In addition, authentication can now be completed online, obviating the need for patients to visit the facility to complete enrollment [29]. Veterans can also now use their [military service] Department of Defense-issued “DS Logon” credentials to log in to My Health*e*Vet and upgrade their account. These changes were made in local settings or by the national program office in response to Veteran and staff feedback about ways to improve enrollment processes obtained through focus groups, online surveys, and quality improvement initiatives. Additionally, strong collaboration between the national program office and VHA researchers continues to inform implementation strategies [9,14,30,31].

### Divergence of Opinions on the Appropriateness of Primary Care for Promoting Personal Health Record Portal Enrollment

National leadership viewed primary care, in its role as the medical home, as the logical place to enroll patients in My Health*e*Vet, but this view differed from patients’ and providers’ perspectives. Both patients and providers stated that primary care should be focused on the already time-consuming demands of providing needed clinical services. Notably absent in our data were patient references to the reorganization of primary care into teams. This may be because the reorganization had occurred in advance of My Health*e*Vet or possibly because patients do not view issues surrounding the structure of their primary care teams as germane to My Health*e*Vet. However, patients found it incongruous to hear about My Health*e*Vet along with clinical issues or vaccines. Likewise, providers did not feel they had time to address issues outside of their clinical demands.

Providers are concerned about the added workload helping patients use patient portals [32]. They are feeling overwhelmed by clinical tasks, including responding to EHR clinical reminders [33], and may not have the capacity to add more to their clinical encounters.

### Provider Ambivalence Regarding the Value of the My Health *e* Personal Health Record Portal

National leaders espoused the importance of primary care providers promoting My Health*e*Vet, but the providers we spoke with described mixed feelings about My Health*e*Vet. Some providers were concerned about what patients might learn by reading their medical record and, therefore, did not encourage enrollment. Yet, as the leaders knew, primary care support may be critical to patient enrollment.

These findings mirror those of others who found poor provider support of patient portals. Witry et al [34] found providers held a limited view of patient health record functions and benefits, whereas Kittler et al [35] found providers are hesitant to electronically communicate with patients. Although such views may be evolving with the spread of PHRs across health care organizations, providers not fully supporting PHRs can still undermine efforts to get providers to promote patient portals. It may be that providers do not see a role for patient portals or how they might fit into their own practice [36]. Provider education, such as an academic detailing approach, may be a way to increase familiarity and interest in My Health*e*Vet by providing tailored and feasible feedback on what My Health*e*Vet promotion could look like in primary care settings [37].

### Lack of Consensus Over Appropriate Patients to Target for My Health *e* Personal Health Record Portal Enrollment

There was some agreement among program leaders and providers that My Health*e*Vet enrollment should be targeted. National leaders felt My Health*e*Vet should be promoted toward patients who had computer access, whereas providers thought about their patients in terms of demographic characteristics, such as age. Regardless of the population of focus, targeting specific populations and monitoring their uptake are effective at increasing patient portal adoption [38], although this may contribute to widening of the digital divide.

### Conclusion

Our findings reveal the importance of seeking a multistakeholder perspective to identify and understand challenges to enrollment in patient portals. More broadly, our findings may have implications for adoption of new patient facing technologies in general. These lessons are important because of the continued trend toward making patient access to care broader (ie, accessible 24/7 asynchronously from any location), the resulting pressures that can surface in the clinical setting as roles shift and adaptation is required, and the implications for resources to support new processes. Implementation strategies will be needed to address these challenges. Additional technologies are being implemented, such as text messaging systems [39-42] and wearable devices [43], both of which will take the time of someone (providers, techs, clerks) to explain to patients what they are, how to use them, and to help them enroll. Similarly, it will be important to bring providers on board for these other technologies because they are likely to be at least partially affected either by the data they provide or patients asking about them.

Our study has several limitations. This work is a snapshot in time, representing the state of the VHA patient portal in 2011-2012. A variety of factors have subsequently influenced the evolution of policies and processes of My Health*e*Vet enrollment. Additionally, this study is limited to the experiences of patients and providers from 3 primary care clinics in the northeastern United States. Although the sites we visited had limited office hours to complete enrollment, this was not uniform across all VHA facilities nationally. Further, our lessons may not be uniformly relevant to other organizations.

Despite these limitations, our findings suggest several lessons for health care organizations seeking to increase enrollment in their patient portals. Although primary care may have seemed an ideal location to promote My Health*e*Vet, and this idea was supported by program leaders, the patients and providers we spoke with did not share this view. In their review of patient portals, Goldzweig et al [44] concluded that additional information about context is necessary to help policymakers better understand how successful portals have been implemented.

Further, our data underscores the importance of speaking to all invested parties. From these 3 critical stakeholder groups—patients, primary care clinicians, and national program leaders—we captured sometimes divergent perspectives regarding how efforts to improve enrollment in the PHR portal aligned with the primary care setting. Although primary care may have intuitively seemed like an ideal setting to improve enrollment, providers and patients offered some cogent reasons that refute this intuitive choice. It was only through our discussions with patients and providers that we learned of their familiarity and existing ambivalence about VHA’s PHR. The state of enrollment was not a reflection of not knowing about My Health*e*Vet, but was instead symptomatic of a system with some obstacles to enrollment and concerns about the role of My Health*e*Vet in primary care. As previously noted, since the time of the study several improvements have been implemented both in marketing strategy and methods, and in the actual enrollment process.

From a sociotechnical perspective, our study raises important questions regarding the relative fit of efforts to increase enrollment in PHR portals within primary care contexts [45]. Primary care providers may not feel it is their responsibility to focus on enrollment and patients may be wary of detracting from issues directly related to their health that are seen as more pressing. Based on our analysis, we recommend that PHR portal enrollment processes be creatively reimagined and streamlined. Patients could, for example, be automatically enrolled unless they opt out, similar to how some organizations structure retirement plans [25]. Proactive, customized implementation strategies, such as those described in the literature, may be considerably effective [46]. Understanding the perspectives that diverse stakeholders may have of such strategies could make all the difference in their success.

## Appendix

Multimedia Appendix 1 Table 1. Principles of Patient-Centered Medical Homes and Exemplary My Health*e*Vet Patient Portal Features.

Multimedia Appendix 2 Focus group and interview guides.

## Abbreviations

EHR: electronic health record
ICT: information and communications technology
PCMH: patient-centered medical home
PHR: personal health record
VHA: Veterans Health Administration
