# Supplementary material for: Encouraging Patient Portal Use in the Patient-Centered Medical Home: Three Stakeholder Perspectives
Source: J Med Internet Res. 2016 Nov 22;18(11):e308. doi: 10.2196/jmir.6488 (PMC5141333; doi:10.2196/jmir.6488)
Supplement: Multimedia Appendix 1 [file jmir_v18i11e308_app1.pdf]

Table 1. Principles of Patient-Centered Medical Homes and Exemplary My HealtheVet Patient Portal Features.

| Patient-Centered Medical Home Principle | Exemplary My HealtheVet Patient Portal Features |
| --- | --- |
| Whole Person. The primary care team is focused on the whole person, with patient preferences guiding care. | Health calendar<br>Health history<br>Health information collection<br>Health screening tools<br>Links to community resources<br>Online courses<br>Track health diaries and measures<br>VA resource information<br>Wellness reminders |
| Team-Based Care. Primary care is delivered by an interdisciplinary team led by a primary care provider using facilitative leadership skills. | Appointment views<br>Caregiver, provider, & health insurance information<br>Secure messaging |
| Enhanced Access. Veterans receive the care they need at the time they need it from an interdisciplinary team functioning at the highest level of their competency. | Appointment views<br>Health screening tools<br>Medication summary<br>Prescription refills<br>Secure messaging<br>Wellness reminders |
| Comprehensive. Primary care serves as a point of first contact for a broad range of medical, behavioral and psychosocial needs that are fully integrated with other VA health services and community resources. | Caregiver, provider, & health insurance information<br>Health calendar<br>Health information collection<br>Health screening tools<br>Links to community resources<br>Online courses<br>Records of care received<br>VA resource information |
| Relationship-based. Every patient has an established and continuous relationship with a personal primary care provider. | Appointment views<br>Records of care received<br>Secure messaging |
| Communication. The communication between the Veteran patient and other team members is honest, respectful, reliable and culturally sensitive. | Health information collection<br>Online courses<br>Prescription refills<br>Secure messaging<br>Wellness reminders |
| Coordination. The primary care team coordinates care for the patient across and between the health care systems including the private sector. | Caregiver, provider, & health insurance information<br>Medication summary<br>Printable VA health summary<br>Records of care received |
