# Supplementary material for: Encouraging Patient Portal Use in the Patient-Centered Medical Home: Three Stakeholder Perspectives
Source: J Med Internet Res. 2016 Nov 22;18(11):e308. doi: 10.2196/jmir.6488 (PMC5141333; doi:10.2196/jmir.6488)
Supplement: Multimedia Appendix 2 [file jmir_v18i11e308_app2.pdf]

Appendix 2. Focus Group and Interview Guides

This is a semi-structured qualitative interview guide. The interviewer may follow-up on issues raised by the participants and probe responses for further information. *Major questions are enumerated; potential probes are given below.

ENROLLED PATIENT FOCUS GROUP

Today, we're going to talk about MyHealth*e*Vet, the VHA's personal health record system. You were all asked to be part of this focus group because you had signed up for MyHealth*e*Vet, and gone through the entire registration process.

1. Can you tell me how you first learned of MyHealth*e*Vet?
2. What do you think about MyHealth*e*Vet?
3. Why do you use MyHealth*e*Vet?
4. Do you have a computer?
5. There are three different levels of MyHealth*e*Vet registration. At the first level, anyone can look at MHV, but the information you see is generic. The second level allows you to put your information in, but you cannot see your personal health record. The third level is "in-person authentication" which allows patients, such as you, to access your personal health record, including your appointments or refilling prescriptions. In order to get this highest level of health record access, you have to come to the VHA and show your identification. I'd like to learn more about your experiences getting full access to MyHealth*e*Vet.
    a. How did you learn about that process?*
        i. Can you walk me through what you did to sign up?; Did you run into any problems?; What could have been better?
    b. Did anyone in your primary care appointments tell you about MyHealth*e*Vet?;
6. What do you think the best ways for us to tell other patients, like you, about MyHealth*e*Vet?
7. Now I want to talk more about the ways the VHA might tell others more about MyHealth*e*Vet and getting full access to it. Imagine you have a primary care doctor's appointment here, at the VHA. What do you think patients should learn about MHV during an appointment?
    a. At what point in your visit do you think would be the best time to have a conversation about MyHealth*e*Vet?
    b. Who do you think would be the best person or people to walk you through this process?
    c. What information do you think is important for them to tell patients?
    d. What should they know about signing up for MyHealth*e*Vet and getting enrolled?
    e. Are there other things we should keep in mind when providers tell patients about MyHealth*e*Vet?
8. Do any of you have any final ideas that you would like to share before we finish?

UNENROLLED PATIENT FOCUS GROUP

Today, we're going to talk about My Health*e*Vet, the VHA's personal health record system, to begin I'd like to learn about what systems you might use to keep track of your health information.

1. Do you currently have a system for keeping track of your health information?
    a. How do you manage your health information, such as your health history, current conditions, medications, and doctor's appointments?
    b. What information is important for you to keep track of?
    c. Do you have a computer?

2. Are any of you familiar with the VHA's patient medical record system, which is call My Health*e*Vet? For those of you who don't know, the VHA has developed a webpage that gives people who use the VHA for healthcare access to parts of their medical record, such as their medications, appointments, and also gives the ability to send messages to your healthcare provider. Have any of you heard of MHV?
3. Now, I'd like to tell you a little more about how MHV works. And then, I want your opinions on how to improve it and tell other patients about it. Description: There are three different levels of MyHealth*e*Vet registration. At the first level, anyone can look at MyHealth*e*Vet, but the information you see is generic. The second level allows you to put your information in, but you cannot see your actual health record. The third level is "in-person authentication" which allows patients, such as you, to access your personal health record, including your appointments or refilling prescriptions. In order to get this highest level of health record access, you have to come to the VHA and show your identification. Have you heard of these levels of registration before now?
    a. [For yes.] What have you heard about getting enrolled?
4. What do you think the best ways for us to tell other patients, like you, about MyHealth*e*Vet?
5. Now I want to talk more about the ways the VHA might tell you more about MyHealth*e*Vet. Imagine you have a primary care doctor's appointment here, at the VHA.
    a. Would you like to learn about accessing your medical record during an appointment?
    b. At what point in your visit do you think would be the best time to have a conversation about MyHealth*e*Vet?
    c. Who do you think would be the best person or people to walk you through this process?
    d. What information do you think is important for them to tell you?
    e. Are there other things we should keep in mind when telling patients about MyHealth*e*Vet?
6. Do any of you have any final ideas that you would like to share?

PRIMARY CARE PROVIDER INTERVIEW GUIDE
I work on a research team that is trying to learn more about the My HealtheVet Clinical Reminder. This is a reminder that appears when primary care clinical staff check in a patient. The reminder prompts the staff to ask the patient a series of questions to determine if s/he is using MyHealth*e*Vet and if not, if s/he might be interested in enrolling. I am speaking with you today to learn how the primary care clinic runs, and within that to figure out good ways to tell patients about.

1. What is your role in patient care?
2. Are you familiar with MyHealth*e*Vet?
    a. What do you know about it?
    b. In general, what are your thoughts about MyHealth*e*Vet?
        i. What do you like about it?
        ii. Do you have any concerns?
        iii. Do you think your patients would be interested in MyHealth*e*Vet?
        iv. Are there particular patients you think would benefit from using MyHealth*e*Vet?, and are there others who you think MyHealth*e*Vet isn't relevant to?
    c. Do you know about the three levels of access to MHV? [Explain 3 levels]
        i. Are you aware of the steps patients need to go through to get the highest access?
    d. Have you had patients ask you about MHV?; Have you helped anyone get access?

3. Aside from what we've already talked about, is there anything else I should know about your experiences with MyHealth*e*Vet?

LEADER INTERVIEW GUIDE

We are working on a project to increase enrollment in MyHealth*e*Vet and getting patients to "In-person authenticate" which means that they will have the fullest access to MHV. We are focusing our efforts on the MyHealth*e*Vet Clinical Reminder that is being used in primary care clinics. Today, I want to learn about your knowledge of [probe according to leader's specific knowledge of and to see what we might learn from your experiences.

[Tailor questions to position.]

1. Can you give me brief description of who you are and what you do?—Something that you might tell someone who is not familiar with VHA.
2. Can you tell me about your experiences and familiarity with MyHealth*e*Vet?
3. What is the relationship between your job and getting patients enrolled in MHV?
4. What are your overall impressions of the efforts to increase MyHealth*e*Vet?
5. Do you have any advice for others who are working on increasing MyHealth*e*Vet enrollment?
6. What do you think about the use of a MyHealth*e*Vet Clinical Reminder in primary care clinics?
7. Do you have other ideas for getting patients enrolled in MyHealth*e*Vet?
8. Aside from what we've already talked about, is there anything else I should know about your experiences?
